# The Deposition and Accumulation of Microplastics in Marine Sediments and Bottom Water from the Irish Continental Shelf

**DOI:** 10.1038/s41598-017-11079-2

**Published:** 2017-09-07

**Authors:** Jake Martin, Amy Lusher, Richard C. Thompson, Audrey Morley

**Affiliations:** 1 School of Geography and Archaeology, National University of Ireland Galway, University Road, Galway, Ireland; 2 School of Biological and Marine Sciences, Plymouth University, Drake Circus, PL4 8AA UK

## Abstract

Microplastics are widely dispersed throughout the marine environment. An understanding of the distribution and accumulation of this form of pollution is crucial for gauging environmental risk. Presented here is the first record of plastic contamination, in the 5 mm–250 μm size range, of Irish continental shelf sediments. Sixty-two microplastics were recovered from 10 of 11 stations using box cores. 97% of recovered microplastics were found to reside shallower than 2.5 cm sediment depth, with the area of highest microplastic concentration being the water-sediment interface and top 0.5 cm of sediments (66%). Microplastics were not found deeper than 3.5 ± 0.5 cm. These findings demonstrate that microplastic contamination is ubiquitous within superficial sediments and bottom water along the western Irish continental shelf. Results highlight that cores need to be at least 4–5 cm deep to quantify the standing stock of microplastics within marine sediments. All recovered microplastics were classified as secondary microplastics as they appear to be remnants of larger items; fibres being the principal form of microplastic pollution (85%), followed by broken fragments (15%). The range of polymer types, colours and physical forms recovered suggests a variety of sources. Further research is needed to understand the mechanisms influencing microplastic transport, deposition, resuspension and subsequent interactions with biota.

## Introduction

Since the onset of the mass production of plastics in the 1940s their durability and utility has made plastics a fundamental component of the modern era. Plastics are robust and have a wide range of uses which has also served to make them one of the most copious forms of anthropogenic marine debris from coastlines to the deep-sea^1^. Millions of tons of lost or discarded plastic items are entering the world’s oceans at a rate that is expected to increase by an order of magnitude by 2025, unless current disposal practices are altered^2^. Microplastics, defined as plastics <5 mm in size, form a numerically dominant component of this anthropogenic debris. These plastics may enter the environment as either primary microplastics, those manufactured to size, or secondary microplastics, which are generated from the breakdown of larger plastic items^3^.

There are a variety of vectors for microplastics to enter marine surface waters and subsequently sink to the continental shelf or abyss. Identified microplastic pathways include sewage systems, riverine inputs, storm water outflows, atmospheric outfall, incorrect disposal, loss during maritime activities, and the *in situ* environmental breakdown of larger plastic items^4–7^. Once in the ocean, plastics can be density modified by processes such as the leaching of additives, biofouling and incorporation within marine aggregates. These processes facilitate microplastics sinking to the seafloor, even if their original densities kept them buoyant^1, 8–10^. The low energy environment, low oxygen levels, cold temperatures and lack of solar UV-radiation in the benthic zone may then slow the breakdown of plastic debris^11^. This could further exacerbate the persistence of microplastics in the marine environment.

The vast expanse of the seafloor has previously been suggested to be an accumulation zone for microplastics^12, 13^. However, the remoteness of the benthic zone has made it an area of limited study. Consequently, microplastic monitoring programs have largely focused on coastal and surface water environments (e.g. ref. 14). Yet, microplastics have been demonstrated to have reached even the most remote deep-sea habitats and have been recovered from at least 2 cm depth from abyssal sediments^8, 12, 15^. Understanding the global distribution of microplastics will be difficult without further analysis of microplastic accumulation on the seabed^16^. While significant attention has been paid to the spatial variability of microplastic deposition between sites, the dynamics of microplastic distribution vertically within the sediment column remain largely unknown^13, 17–21^. Trawling, bioturbation, tidal forcing, and weather events can all influence the distribution of particles and litter within marine sediments and within the overlaying water column^22–24^. The fraction of microplastics readily suspended within the benthic section of the water column remains to be quantified. Such resuspension events can represent a pathway for the repeated exposure of filter feeders to previously deposited microplastics^25^. Further, Taylor, *et al*.^26^ found that deposit feeders might ingest even greater quantities of microplastics than suspension feeders. These factors could make the benthic zone an environment with high potential for facilitating microplastic uptake by organisms.

Benthic habitats in coastal settings that support a diverse array of organisms (including commercially targeted species) and biota could be susceptible to intensified microplastic accumulation due to enhanced maritime and commercial fishing activities. Close proximity to the increasingly common anthropogenic stressors of coastal industrialisation and population growth may also contribute to enhanced microplastic deposition in these locations^7^. For example, the Aran Grounds fishery, located off the west coast of Ireland outside Galway Bay, produced a *N*. *norvegicus* catch of approximately €95 million in 2014, supporting the entire fishing fleet based in Rossaveal^27^. *N*. *norvegicus* have been demonstrated to consume microplastic fibres and these stocks may be at risk of biological impacts^28^. A range of potential impacts from microplastics have been suggested for various marine biota including: inflammation, endocrine disruption, liver toxicity, carcinogenesis, reduced fitness and reproductive failure^3^. However, laboratory-based studies often employ microplastic concentrations far exceeding environmental levels and are therefore difficult to equate with the health of wild populations^13^. Identifying accumulation hotspots and quantifying environmental concentrations for microplastics along the seafloor is therefore critical for understanding the health of marine ecosystems in support of the Marine Strategy Framework Directive (MSFD) (2008/56/EC) and other initiatives addressing marine pollution.

Plankton records indicate a significant increase in the abundance of microplastic pollution from the 1960s–1990s within North Atlantic surface waters^29^. A record of Belgian beach accumulation shows the same trend of increasing pollution, with an almost tripling in microplastic concentrations from 1993 to 2008^19^. Establishing the fraction of microplastics accumulating in the benthic zone is important as plastic density, spatial usage and transport via currents could lead to differing microplastic legacies in different environments. Nylon (polyamide, PA) concentrations observed in beach and surface waters are below expected levels based on maritime usage, particularly fishing activity. It has been proposed that PA materials are settling out offshore^11^. However, to our knowledge no attempt to create a depositional history for microplastics in marine sediments has been undertaken; nor have microplastic concentration trends been established for the duration of the mass consumption of plastics since the 1940s, in any environment.

The aim of this study is to provide the first assessment of microplastic deposition in sediments collected from the Irish continental shelf and within their overlaying water-sediment interface. More specifically, this study investigates the history of microplastic deposition on the seafloor and examines how sedimentation rates, proximity to anthropogenic activity, and disturbance regimes may impact microplastic distribution between sites and within the sediment column.

## Methods

### Study Areas and Oceanographic Setting

Two main study areas on the Irish continental shelf were selected to examine microplastic deposition in remote and proximal oceanographic environments. Remote locations (R) include two sites in Blacksod Bay, County Mayo, and three offshore stations near the continental margin, approximately 70 kilometres northwest of the Inishkea Islands. Blacksod Bay is characterised by rural coastal settlements and limited fishing activity. The two samples from Blacksod Bay were collected in shallow (10 and 31 m water depth) high-energy depositional environments. Except for these two samples (R02 and R07) all remaining sample locations are below the seasonal mixed layer depth (MLD) where inter-annual atmospheric forcing (e.g. wind and rain) do not alter depositional regimes. Remote sites, R09, R10, and R11 are located on the continental margin just south of the Donegal-Bay Canyon head, which places them within the Shelf-Edge Current (SEC). Mean bottom current speeds of the SEC at these sites lie between 10–20.ms^−1^ ^30^. The sandy nature of surface sediments at these sites suggests a high energy sedimentation regime. This is not surprising since submarine canyon heads such as the Donegal-Bay Canyon can act as an area of intensive sediment transport channeling large volumes of suspended materials from the shallow shelf into the deep ocean^31^.

Proximal sites (A) focus on the Aran Grounds fishery (five sites) situated west of the Aran Islands and an additional site in the North Sound of Galway Bay in between Rossaveel and Inishmore. These sites are characterised by bottom trawl fishing (e.g. *Nephrops norvegicus*), utilizing a variety of plastic polymer gear types. They are also in close proximity to urban and industrialized areas. At the surface the Irish Coastal Current (ICC) that flows along the Irish Coast from South to North dominates the hydrography of the Aran Grounds fishery^32, 33^. On its way northwards, the ICC transports fresh riverine outflow waters from the Shannon Plume towards the fishery^34^. During the winter when river discharge is enhanced^35^ the sediment load of the Shannon plume is enhanced contributing to sediment accumulation in this area. An anticlockwise circulation characterizes the surface flow within Galway Bay with ICC waters entering the Bay via the South Sound. In the inner bay these waters mix with the discharge of the River Corrib. The outflow of this river passes through the urban environment of Galway City and then travels westward, parallel to the north shore, and exits the bay through the North Sound. However, the recirculating pattern of river water in the vicinity of the sound extends the residence time of water within the bay and leads to a build-up of materials in the outer bay^36^.

### Sample Collection

Sediments from remote locations were retrieved during a R.V. Celtic Explorer cruise (CE14019) on the 15^th^ and 16^th^ of February 2014. The Aran Grounds fishery and the fishery within Galway Bay’s North Sound were sampled during a R.V. Celtic Voyager cruise (CV15025) between the 7th and 9th of October 2015. We acknowledge that samples were taken 19 months apart and that this may affect sedimentary and microplastic deposition for sites that are located above the MLD. However, of all stations analysed only two (R02 and R07) are located above the MLD. We therefore do not think that this temporal offset in sampling is likely to be a major contributor to plastic accumulation rates for most samples analysed here. For each location, two core liners of 66 mm diameter were carefully placed into a reineck box corer in order to sample and preserve the water-sediment interface with both replicates. All sampled cores were subsequently transferred to the National University of Ireland, Galway (NUIG) and refrigerated in an upright position until processed.

### Procedural Contamination Controls

To eliminate post-depositional contamination strict controls were followed during sample collection and preparation. Nitrile gloves, cotton clothing and laboratory coats were worn during the handling of samples. Glass, metal and cardboard equipment was used whenever possible. Where plastic equipment was used the polymer structure of the equipment was controlled for within the study using Fourier Transform Infrared Spectroscopy (FT-IR). Sterile consumables were used directly from packaging. All equipment and laboratory surfaces were cleaned with compressed air, natural fibre brushes or deionized (DI) water and inspected for procedural contamination between subsample examinations. Potential airborne contamination was monitored using routine 24 h exposures of filter paper pads, as well as filter paper pad exposures during laboratory work. Airborne fibres had their physical characteristics noted and their typology was excluded from any samples processed within a 72 h period surrounding airborne fibre identification. Contaminant compositions were controlled for using FT-IR. Equipment and samples were kept covered whenever possible. This included sealing sieves between and during use. The precautionary principle was employed during this study and any debris that was deemed to be possible procedural contamination was rejected from the study. Rayon was excluded from this study, as it is difficult to distinguish from cellulosic materials naturally occurring within the seabed.

### Core Processing

Cores were processed in a randomly selected order to prevent bias in the sampling extraction phase. When present, the water-sediment interfaces were siphoned from cores and vacuum pumped onto glass microfiber papers (GF/C). Siphons were flushed with DI water to extract microplastics that may have adhered to the tubing. Filter papers were stored in sealed petri-dishes until inspection. Sediments were sliced at 0.5 cm intervals using a metal blade. For each core, alternating depth intervals (0.0–0.5 cm, 1.0–1.5 cm, 2.0–2.5 cm, 3.0–3.5 cm, 4.0–4.5 cm), as well as the deepest depth (core dependent) were isolated and disaggregated using a Stuart SSL1 orbital shaker. Sediments were shaken for up to 48 h in sealed glass Erlenmeyer flasks. The fine fraction of the sediments was then filtered out with DI water over a 63 μm sieve. The coarse fraction of the sediments (>63 μm) was dried for 24 h under a contamination-controlled tent. Dry sediments were stored in sterile sealed glass vials until inspected for microplastics.

### Microplastic Extraction

Two extraction methods were compared for developing a protocol for recovering microplastics from sediment cores. Dense media floatation using sodium polytungstate (SPT) (Na_6_H_2_W_12_O_40_) was compared against dry sieving of sediment subsamples. Dense media floatation of microplastics was conducted under a fume hood by submerging a 63 μm mesh net containing the coarse fraction of a subsample into a 1.65 g/ml solution of SPT. Floating microplastics were then extracted from the surface of the dense media using a ladle. For the second method, interlocking sealed stainless-steel sieves of different mesh sizes (500 μm, 400 μm, and 250 μm) were used to dry-sieve sediment samples in manageable quantities. Sieves were initially shaken for approximately 60 seconds and re-shaken for approximately 5 seconds between inspections of size intervals. The size range of plastics examined was between 5,000–250 μm. The lower size limit of 250 μm used in this study represents the mesh size used for the study of microplastics in the surface waters of the NE Atlantic^14^.

### Microplastic Identification

**Figure 1:**
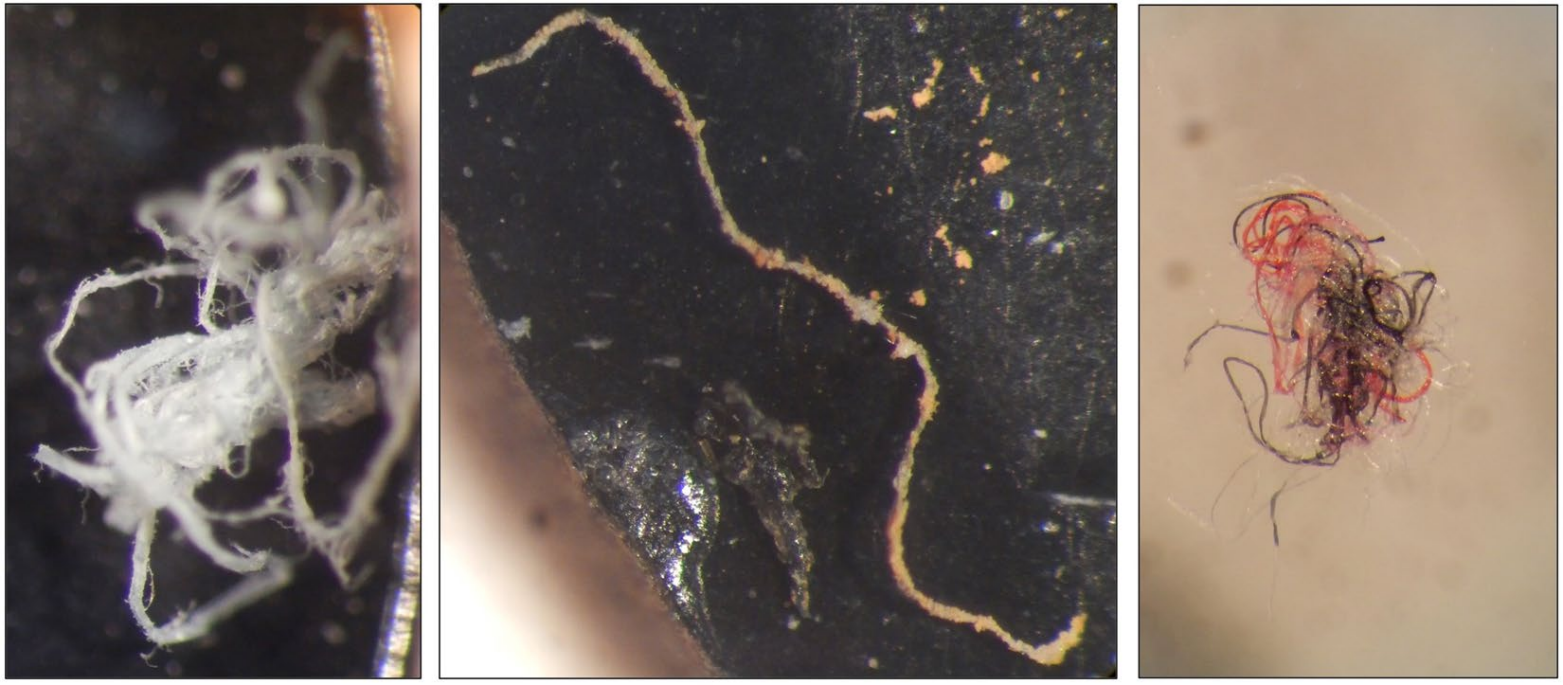
A subset of recovered microplastics at 40–50x magnifications. (L) A frayed and tangled fibre from Galway Bay’s North Sound. (C) A heavily biofouled transparent fibre from the Aran Grounds. (R) A tangled ball of fibres identified during method testing from Galway Bay’s South Sound (52°57.722N, 9°33.358W).

All recovered microplastics were inspected under a Leica Wild M8 stereomicroscope at magnifications up to 50x (Fig. 1). Only microplastics of sizes 250–5000 μm were identified and counted according to protocols developed by Lusher, *et al*.^33^ for studies where FT-IR is not readily available. This larger size range of microplastics was chosen as microdebris of this size is more likely to be correctly identified (visually) as plastics than smaller plastics. In addition, this larger size fraction of plastics is also less likely to be affected by changing bottom water current regimes or other processes that may disturb the water-sediment interface as fragments/fibres are comparable to or larger than the sediment sizes of samples analysed here. Potential microplastics were sorted into fragments and fibres. Unnatural colours and/or shininess were used as indicators of potential microplastics. Particles that did not possess uniform colouration, were matt, or had potentially cellular or organic structures were rejected. Fibres were also checked for three-dimensional bending and uniform thickness and rejected otherwise. A double ID system was used with two experienced researchers confirming recovered debris as potentially microplastics through visual analysis. A Bruker vertex 70 Fourier Transform infrared Spectrometer with an accompanying Bruker Hyperion 1000 Microscope was used for analytical confirmation of the material composition of microdebris and for the indexing and control of potential contaminants using the OPUS polymer database. For FT-IR analysis a random subsample of plastics was chosen from each layer of contaminated plastics. Microdebris were flattened using a diamond compression cell to maximize the resolution of FT-IR readings. Slides were cleaned with alcohol between processing of individual debris to ensure an uncontaminated reading. Particles with (<0.7) Euclidian Distance (ED) matches to known polymer compositions were accepted as plastics following the procedures of Lusher, *et al*.^38^. The majority of microplastics accepted without FT-IR analysis (n = 25) were visual matches (colour and physical form) to FT-IR confirmed microplastics from the same subsample or corresponding replicate.

### Distribution Analysis

All statistical analyses were performed using Microsoft Excel and SPSS. The abundance, location, typology, colour and polymer structure of microplastics were investigated. Data from contiguous replicates was combined into a single station profile. Microplastic counts for these profiles were then standardised to 26 ml per sediment layer, per station for comparative statistics ((# plastics ÷ subsample volume) × 26). Standardisation to 26 ml provided the minimal amount of raw data manipulation necessary for comparative purposes, as it was the most common combined subsample volume. Water-sediment interfaces ranged from 30 ml to 290 ml after corrections for the flushing of DI water through the siphon. Water-sediment interfaces were analysed without standardisation based on the assumption that they drained through sediment samples at an uneven rate during processing and that no microplastics were lost during this process.

The resuspension of particles is a dynamic process in the natural environment and it can be assumed that the transport, storage and processing of replicate cores impacted the number of microplastics in suspension at the time of extraction. As cores were carefully handled it was assumed that any particles brought into suspension by the sampling process could have been suspended by minor natural disturbance and that any particles that settled out may have done so under low energy conditions on the seafloor. Water-sediment interfaces and superficial sediments are therefore assumed to be representative of natural conditions within the remit of the study.

Independent t-tests were performed where conditions for normality and homoscedasticity were met. Where data was not normally distributed non-parametric statistical tests were performed. Independent t-tests were used to examine potential differences in total microplastic abundances in cores between the fisheries stations and remote stations. A potential relationship between corrected water-sediment interface volumes and their total microplastic loading was assessed using Spearman’s ρ. Potential relationships between the distribution of microplastics within the cores based on sediment depth and sedimentation rates were also assessed using Spearman’s ρ. An alpha level of 0.05 was used for all statistical tests.

### Age determination of samples

To assess degree of microplastic burial within the sediment column after deposition all sediment cores used in this study (except R02) were dated using AMS C^14^ dating of carbonate organisms from the deepest available sediment sample for each core. The deepest/oldest sediments were dated in order to avoid contamination of bomb carbon 14 (post 1960). Samples were processed at the UCI Keck Carbon Cycle AMS Program at the University of California, Irvine, USA. A minimum of 2.5 mg of carbonate shells were sent for dating from each subsample dated. The median weight of carbonate organisms picked for dating was 5.1 mg per subsample. Only the largest planktonic or epifaunal species, with undamaged shells, that exclusively lived within the top surface sediments of the seafloor were accepted for carbon dating. Results from AMS C^14^ analysis were processed using Calib Rev 7.0.2 software and the MARINE 13 calibration dataset^39, 40^. The implicit reservoir age correction (ΔR = 0) for all dates was applied, as the precise reservoir correction for western Ireland is uncertain. The weighted mean average (WMA) of the calibrated probability distribution for each age with their respective 2σ confidence ranges are reported in Supplementary Table S1. To derive the age of sediments at the maximal microplastic burial depth for each site as well as sedimentation rates a linear regression model of core depth over the WMA age was used assuming that surface sediments are modern. We are confident surface sediments are modern, since the water-sediment interfaces for each of the cores were intact when sampled.

## Results

### Method Validation

Recovery rate tests performed on 60 spiked subsamples produced an average first round recovery rate of 89.0% using the sieving method. An average recovery rate of 54% was achieved for SPT floatation from 5 spiked subsamples. The SPT flotation method was therefore considered inferior in the available laboratory setting for plastics with a size range of 5,000–250 μm and discontinued in favour of the dry sieving method.

### Contamination Control

27 fibres were identified by FT-IR as cellulosic (possibly rayon) within sediment samples (ED range = 0.161–0.661). 24 of these fibres lacked any form of distinguishing pigmentation. Single instances of red, black and blue rayon/cellulose were recorded. Analysis could not exclude potential natural sources, e.g. seaweed, for the 27 fibres. A single airborne particle identified within the laboratory produced a weak plastic polymer signature (Acrylic, ED = 1.05). This signature was outside of the acceptable confidence level for plastic polymer origin used within the study. The airborne particle did not match the single acrylic microplastic found at station R11. The remaining airborne fibres chemically controlled for using FT-IR (n = 4) showed strong signatures for cellulosic origin (ED range = 0.326–0.335). Therefore, any particles classified as cellulosic (possibly rayon) were excluded from microplastic counts.

### Microplastic Characterisation

All microplastics identified were secondary microplastics, either fibres (85%) or fragments (15%) and exclusively fibres were recovered from water-sediment interfaces. No spheroids, pellets or films were visually or chemically identified within the study. The most frequent microplastic colour observed for all depth intervals studied was blue (29%), followed by transparent (21%), white (16%), red (16%), black (12%), green (3%) and grey (3%). For water-sediment interfaces, only blue (72%) and red (28%) fibres were observed. Blue, grey and black microplastics were only observed within fishery sediments, while green microplastics were only observed within Blacksod Bay.

A subset of recovered microplastics (n = 24, 39%) were FT-IR tested for confirmation of polymer identity. Four polymer types were identified within the study. 23% of microplastics recovered from cores were confirmed as polyamide (ED range = 0.313–0.641), 11% as PET (ED range = 0.269–0.416), 3% as polypropylene (ED range = 0.270–0.490) and 2% as acrylic (ED = 0.486). In Table 1

**Table 1:** FT-IR confirmed polymers by station and burial depth.

**Polymer**	**Stations**							**Maximum Depth (cm)**
	**A01**	**A06**	**A07**	**A08**	**A13**	**A14**	**R11**	**All Stations**
**Polyamide**	***4***	***4***	***2***	***1***	***3***			**2.5** ± **0.5**
**Polypropylene**	***2***							**3.5** ± **0.5**
**PET**	***1***			***1***	***1***	***1***	***3***	**2.5** ± **0.5**
**Acrylic**							***1***	**2.5** ± **0.5**

, FT-IR analysed microplastics are reported by station along with the maximum burial depth observed for each polymer type. The remaining 61% (n = 38) of microplastics were accepted solely through visual identification and physical manipulation. As mentioned above the majority of microplastics accepted without FT-IR analysis (n = 25) were visual matches (colour and physical form) to FT-IR confirmed microplastics from the same subsample or corresponding replicate. >12 microplastics, predominantly fibres, were lost either during extraction, manipulation or transport to the FT-IR facility and were unavailable for spectral analysis. 4 microplastics were too small for a clear FT-IR reading to be obtained.

### Microplastic Distribution

**Figure 2:**
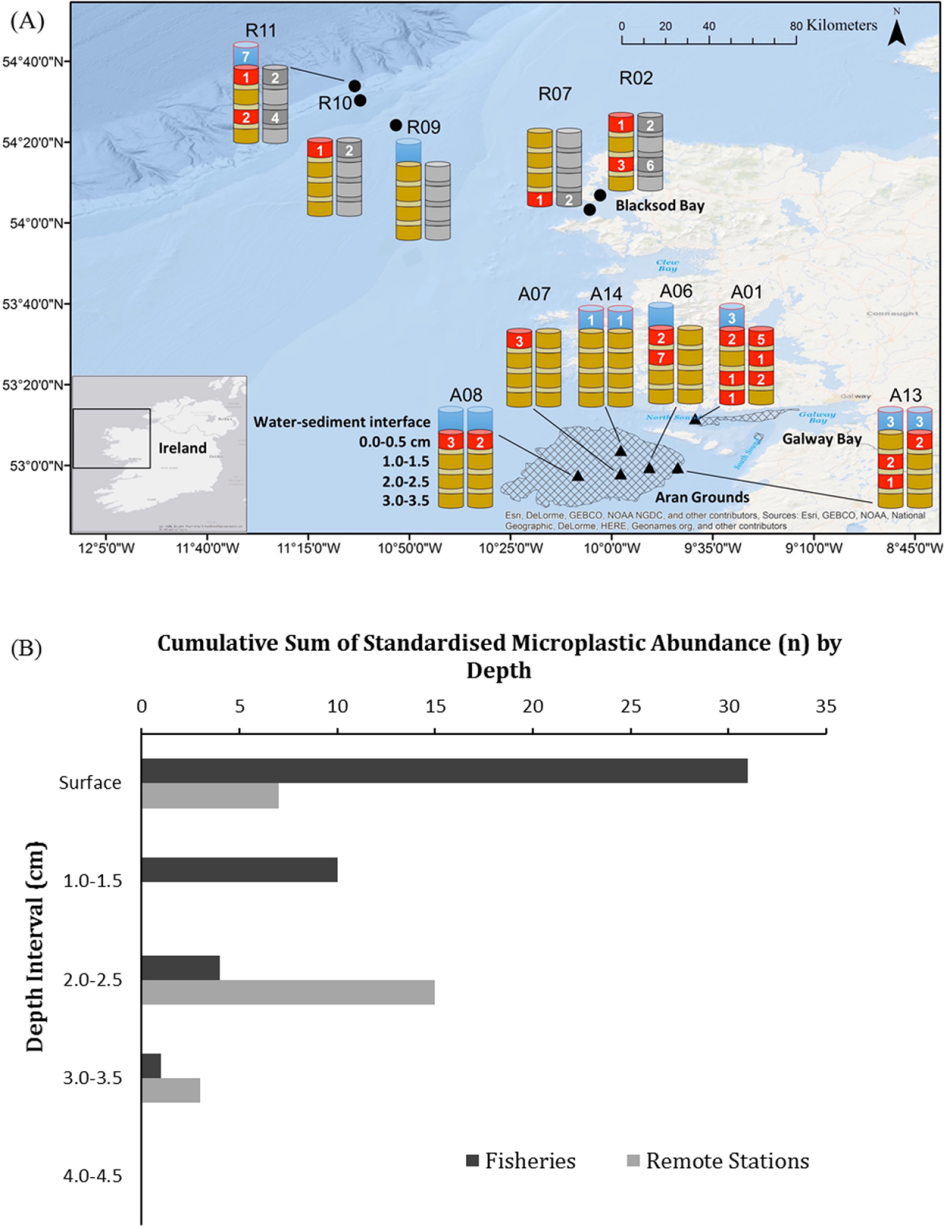
Locations of sample sites and microplastics distributions (**A**). Hatched areas designate *N*. *norvegicus* habitat (data provided by Marine Institute Ireland). Fisheries stations are shown as triangles and remote stations as circles. Core replicates are shown at their alternating inspection intervals to the maximum depth of microplastic burial observed within the study (3.5 cm). Blue boxes represent water-sediment interfaces and red boxes represent contaminated sediment subsamples. Microplastic counts are shown within the boxes. Where replicates were not available they were represented through volume standardisation, shown here as the grey cores. (**B**) A depth profile of standardised microplastic counts shows the vertical distribution of contamination within the two study areas. Maps were produced in ArcGIS ESRI version 10.3. using the Ocean Basemap (http://goto.arcgisonline.com/maps/Ocean_Basemap). The main base map of Ireland was selected from within ArcGIS and the smaller insert map of Ireland was taken from OpenStreetMap contributors available under the Open Database Licence at www.opendatacommons.org/licenses/odbl.originates.

One whole replicate and one replicate aliquot were examined for each fisheries station. Silts and muds dominated the Aran Grounds, while the samples from outer Galway Bay contained a mix of mud and sand with high organic content. Due to logistical constraints single replicates were examined for remote stations. Blacksod Bay was characterised by sandy sediments, while fine Holocene mud was predominant at offshore stations. A total of 62 microplastics were recovered from the 11 stations (including replicate cores) at alternating 0.5 cm depth intervals to a depth of 3.5 cm (Fig. 2). Microplastic count data was standardised to the most common volume for individual sediment layers at a station for comparison between sites.

Of the 62 recovered microplastics, 18 were identified from 6 (of 10) water-sediment interface samples (7 of 11 stations) and 44 microplastics were identified within 85 sediment subsamples from 12 of 17 examined replicates (10 of 11 stations). A sharp decrease in the total microplastic count with sediment depth was observed. 23 microplastics were found in the top 0.5 cm of sediment, 10 microplastics in the 1.0–1.5 cm sediment layer, 9 microplastics in the 2.0–2.5 cm sediment layer and 2 microplastics in the 3.0–3.5 cm sediment layer. Remote stations did not exhibit the trend of a decrease in microplastic counts with sediment depth when examined as an individual case. Depth interval examination ceased after the 4.0–4.5 cm layer, as no microplastics were recovered at any station at this depth.

The water-sediment interface and the top 0.5 cm of sediment contained 66% of all recovered microplastics. The water-sediment interfaces and top 2.5 cm of sediments contained 97% of all recovered microplastics.

Volume standardisation of samples produced a mean of 7.67 ± 2.09 microplastics per station for the Aran Grounds and Galway Bay (A) and a mean of 6.33 ± 4.91 microplastics per station for the remote areas (R). This places both areas examined within error of each other in terms of total microplastic abundance. Microplastics were found at all stations except R09, which was the station furthest removed from zones of intensive material transport within the study^31–33^. Station A01 contained the most polymer varieties confirmed within a single station (n = 3) and had the largest abundance of recovered microplastics (n = 15). However, station R11 had the largest abundance of microplastics (n = 16) after standardisation of sample sizes.

### Distribution Analysis

An independent t-test indicated no statistically significant difference in total microplastic abundance within cores between fisheries and remote stations (t = 0.241, p = 0.815). However, an intra-fisheries independent t-test between Galway Bay and the Aran Grounds indicated a statistically significant accumulation of microplastics within Galway Bay, as opposed to the Aran Grounds, although this was determined from a limited number of samples (t = 2.475, p = 0.033) (Fig. 2). No statically significant correlation could be found between the volume of a water-sediment interface and microplastic abundance (Spearman’s ρ, r = 0.456, p = 0.185).

For all stations increasing sediment depth was highly correlated with a decrease in microplastic contamination (Spearman’s ρ, r = −0.459, p = 0.01). However, no statically significant correlation could be found between the estimated sedimentation rate of a station and the abundance of microplastics within the examined sediment layers at that station (Spearman’s ρ, r = 0.411, p = 0.238). Nor could a statically significant correlation be found between estimated sedimentation rates and the depth of microplastic burial (Spearman’s ρ, r = 0.492, p = 0.148).

### Age of sediments

To assess the degree of post-depositional transport of microplastics in the sediment column we report the maximum age of sediments in which microplastics were found for each station in Tables S1 and S2. Four out of 11 stations (A01, A06, A13, and R11), have maximal burial depth of microplastics that pre-date (±2σ) the onset of plastic production in the 1940s (Table S2). Two of these, A06 and A13, from the Aran Fishing grounds have plastics deposited in the sediment layer below modern sediment (e.g. within 1 ± 0.5 cm), while the other two, A01 and R11, have the lowest accumulation rates recorded here and plastics were found within 3 ± 0.5 cm and 2 ± 0.5 cm of the modern layer respectively (Table S2). Station R02 from Blacksod Bay was collected from very shallow waters at a depth of 10 m, where the sediment column is highly susceptible to redistribution. This station is within the MLD where it is subject to seasonal weather events (e.g. storms), waves, and tides that are able to relocate the top layers of sediments. Ages for burial depths and sedimentation rates were thus not determined for this location and the entire sample is considered ‘modern’ (e.g. post 1950) based on the prevailing oceanographic conditions at the station. The consistency between ages for each region and the absence of age reversals or sudden increases in dates strongly indicates that sedimentation rates for all stations are constant at least for the top 20 cm of each core.

## Discussion

Results indicate that microplastics have become pervasive across the Irish continental shelf. Moriarty, *et al*.^41^ found fishing intensity accounted for only 11% of observed associated debris distribution on the Celtic Sea seafloor. This weak relationship was attributed to the spreading of debris by the complex oceanographic processes of the region. The results of this study demonstrate that this trend in litter dispersal extends into the micro-metre scale on the Irish continental shelf.

The variation in microplastic abundance between individual stations, but not across larger areas is similar to the findings of Alomar, *et al*.^20^. This highlights the need to understand microplastic accumulation processes at a higher resolution than the regional scale. Further the fragmented forms of the recovered microplastics indicate that the breakdown of larger plastic items into secondary microplastics is the primary source of microplastic contamination on the Irish shelf. An abundance of blue microplastics and fibres have previously been identified in Irish surface waters^14^. These typologies were also prevalent in sediment and bottom water samples collected here, suggesting that the entire water column may be contaminated with these types of microplastics.

Microplastics were prevalent in water-sediment interfaces and superficial sediments suggesting significant exposure potential for both filter and deposit feeders (e.g. sea pens and sea cucumbers)^26^. The large accumulation of microplastics at Station R11, especially within the water-sediment interface, may be the result of shelf edge oceanographic forces near the margins of the Rockall Trough. Canyon features may serve to make shelf break zones microplastic hotspots. This would create a heightened microplastic exposure risk on the shelf slope, despite being far removed from source points.

Although microplastics were found to be limited to the top 3.5 ± 0.5 cm of sediments, their distribution pattern within the sediment column made sampling depth an important variable in depositional analysis. Bulk superficial samples are routinely inspected for microplastics at a variety of sediment depth intervals, e.g. 0–1 cm, 0–3.5 cm and 0–5 cm^8, 12, 20, 42^. The application of these sampling ranges to the cores presented here would produce a large variation in final microplastic counts (Fig. 2). The expression of microplastic concentrations, whether performed as a function of volume or weight would then also be affected. This represents a potential obstacle in inter-study comparison or in quantifying total microplastic abundance in the benthic zone. Therefore, sediment-sampling depth is an important consideration in the development of any future monitoring programs. The results of this study indicate that a minimum of the top 4 cm of the seabed should be sampled for adequate abundance data to be collected on microplastics. A standard 5 cm sample would represent a more precautionary approach. However, a case-by-case decision is most appropriate as sampling protocols should be reflective of a site’s potential for microplastic burial.

The assessment of post-depositional transport of microplastics in the sediment column reveals that the majority of microplastics were found in modern sediments (e.g. post-date onset of plastic production). For only four out of the eleven stations analysed the maximum burial depth for microplastics predates the onset of microplastic production in the 1940s and the maximum burial depth for two of these is within the layer following the modern sediment layer. For these stations we therefore infer that bioturbation and/or physical disturbances (e.g. by fishing gear) that transport microplastics down into the sediment column is minor. It is noteworthy that the oldest ages for maximum burial depths of 1263–1044 CE and 778–549 CE also correspond to sites that have low sedimentation rates (e.g. 208 and 191 years per 5 mm respectively). These ‘old’ sediments are still within 2 and 3 ± 0.5 cm of the modern layer respectively. For sites analysed here, results therefore suggest that maximum burial depth of microplastics is generally confined to modern sediments.

Following, none of the stations examined here showed signs of large-scale disturbance and bioturbation events as observed in other regions of the Irish Shelf ^24^. If large-scale disturbances occurred they did not appear to significantly influence either microplastic or carbonate shelled epifauna distributions. Further, the statically significant trend of a decline in microplastic abundance with sediment depth in the cores of this study suggests that no significant microplastic accumulation or disturbance occurred below the study cut-off point of 4.5 cm. This trend also indicates that microplastic deposition may be increasing over time. However, remote stations did not exhibit the pattern of an overall decrease in microplastic abundance with sediment depth as observed at fisheries stations. This may be a product of the greater variability for microplastic counts at these locations, a higher sedimentation rate within Blacksod Bay (e.g. R07) and/or possible sediment mobility within the energetic environment of the Bay. The existence of a shallow layer of microplastics within superficial sediments aligns with the relatively low sedimentation rates of the study stations. However, the absence of a correlation between projected sedimentation rates and microplastic abundance indicates microplastic dispersal in this area is predominantly governed by other oceanographic mechanisms than sediment outfall.

It is important to highlight that this investigation focused on the distribution of microplastics in the size fraction >250 μm. Results presented here should therefore not be considered as quantifying the total standing stock of microplastics at specific sites. In addition to using targeted sections of the cores a large proportion of recovered microplastics in other sediment studies have been smaller than the 250 μm size limit used in this study^8, 42, 43^. Therefore, microplastic counts presented here may considerably under represent total microplastic contamination within examined subsamples. All recovered cellulosic fibres were also discounted from this study, as a clear distinction could not be made between natural cellulosic forms and chemically engineered cellulosic forms.

Despite the limited number of FT-IR confirmed polymer compositions (n = 24), the large proportion of PA microplastics within this subset (59% of confirmed polymers) supports the hypothesis that the seafloor is a significant accumulation zone for this form of debris^11, 19^. This proportion of PA far exceeds average global coastal contamination levels (≈3%)^18^. A maritime discharge directly into the study area, e.g. sourced from common fishing gear, and/or an irregular land-based contribution could produce this effect^11^. Microfibers within *N*. *norvegicus* specimens from the Clyde Sea have previously been analytically linked to fishing gear type materials^44^. The neutral to negative buoyancy of PA may also lead to its accumulation at depth^4^. PA fibres have also previously been found within the gut contents of *N*. *norvegicus* from both the Mediterranean and Atlantic^44^. In Welden and Cowie^28^ PA represented 37.2% of the microplastics present within the gut contents of *N*. *norvegicus* from Scottish fisheries. The high concentration of nylon micro-fibres ingested by *N*. *norvegicus* from several areas implies that fisheries may be experiencing high exposure to this form of microplastic debris at an international scale. Results here show that the microplastic loading within *N*. *norvegicus* habitat in Ireland closely aligns with microplastic types typically documented within *N*. *norvegicus* gut contents. This highlights the need for laboratory-based experiments on the physiological effects of microplastics that are representative of environmental concentrations.

The presence of PA, polypropylene and PET at proximal station A01 suggests this station has become an accumulation zone for a variety of degraded plastic materials. A statistically significant accumulation of microplastics at station A01 as compared to other stations may be a product of compounding factors including the narrow geometry of the North Sound, its proximity to a developed coastline and water recirculation within Galway Bay^19, 28, 36^.

## Conclusion

A shallow layer of microplastics has formed along the Irish seafloor within the top 3.5 ± 0.5 cm of sediment and within the overlaying bottom water. While average microplastic abundances for the 5 mm−250 μm size fraction were similar across the entire area of the continental shelf examined, individual stations showed wide variation in microplastic abundance. Accounting for the fraction of microplastics <250 μm in size could potentially reveal an even greater presence of contaminants in this region than is documented here.

The vertical profile of microplastic distribution within the sediment column was demonstrated to be an important factor in determining environmental concentrations. The statistically significant trend of a rapid decrease in microplastic abundance with sediment depth observed within the fisheries near Galway supports the assumption that microplastic deposition is increasing over time. Future sampling protocols for quantifying microplastic presence in the seabed should account for microplastic burial potential at a site. A minimum of a 4–5 cm core is recommended for seabed studies based on results presented here.

The polymer varieties of microplastics recovered during this study are commonly employed in both maritime and land-based activities. Further investigation is required to understand the transport of microplastics within marine ecosystems and any environmental consequences. Further studies are also required to continue to assess the build-up of specific microplastic forms in different environmental compartments and to model microplastic transport pathways and address source points.

### Data availability

The datasets generated during and/or analysed during the current study are available from the corresponding author on reasonable request.

## Electronic supplementary material

Supplementary Information
