# Supplementary material for: The Deposition and Accumulation of Microplastics in Marine Sediments and Bottom Water from the Irish Continental Shelf
Source: Sci Rep. 2017 Sep 7;7:10772. doi: 10.1038/s41598-017-11079-2 (PMC5589889; doi:10.1038/s41598-017-11079-2)
Supplement: Supplementary file 1 — Supplementary Information [file 41598_2017_11079_MOESM1_ESM.pdf]

Supplementary Information to:

The Deposition and Accumulation of Microplastics in Marine Sediments and Bottom Water from the Irish Continental Shelf

Submitted to Nature Scientific Reports

Jake Martin[1], Amy Lusher[1], Richard C. Thompson[2], Audrey Morley[1*]

[1]School of Geography and Archaeology, National University of Ireland Galway, University Road, Galway, Ireland

[2]School of Biological and Marine Sciences, Plymouth University, Drake Circus, PL4 8AA, UK

*Corresponding Author: audrey.morley@nuigalway.ie

Table S1: Carbon dated depth intervals, epifauna used and AMS C^{14} results.

Lab ID*	Core	Depth (cm)	Species	^{14}C age (BP)	±1σ	WMA (cal yr BP)	2σ range	Sedimentation rate/5mm	Reference
169779	A01	18	*T. communis*	6980	20	7477	7423-7539	208	*This Study*
169781	A06	12	*Q. semulina, A. beccarii*	2170	15	1764	1695-1831	73	*This Study*
169782	A07	14	*H. balthica*	1565	15	1125	1051-1187	40	*This Study*
169783	A08	20.5	*Q. semulina, A. beccarii*	2820	15	2587	2486-2680	63	*This Study*
169785	A13	11	*T. communis, C. striatula, Q. semulina, A. beccarii, Gastropod, Eulimidea (sp.)*	1310	15	854	789-910	39	*This Study*
179348	A14	15.5	*Q. semulina, H. balthica*	1565	20	1125	1050-1194	36	*This Study*
179352	R07	16	*Q. semulina*	760	20	401	320-465	13	*This Study*
179351	R09	15	*G. bulloides, N. pachyderma, H. balthica, A. beccarii*	3885	20	3857	3771-3947	129	*This Study*
179349	R10	14	*Q. semulina, H. balthica, G. bulloides*	2690	25	2384	2312-2470	85	*This Study*
179350	R11	20	*G. bulloides*	7170	60	7640	7525-7771	191	*This Study*

* UCI KECK Carbon Cycle AMS Program at the University of California, Irvine in the USA

Tabel S2: This table provides an approximate age range (±2σ) for each sample depth. Black font indicates depths/ages where microplastics were present and shaded cells indicate which sediments are considrered modern (post 1940).

Core	Depth [cm]	WMA [cal yr BP]	2σ range [cal yr BP]		water-sed. interface	Age (±2σ) [CE] vs. Depth [cm]									
						0	0.5	1	1.5	2	2.5	3	3.5	4	4.5
A01	18	7477	7423	7539	>1950	2015	1806	1603	1387	1190	968	778	549	365	130
A06	12	1764	1695	1831	>1950	2015	1939	1874	1786	1733	1634	1591	1481	1450	1328
A07	14	1125	1051	1187	*na*	2015	1973	1940	1888	1865	1803	1790	1718	1715	1633
A08	20.5	2587	2486	2680	>1950	2015	1950	1894	1819	1772	1688	1651	1557	1530	1427
A13	11	854	789	910	>1950	2015	1974	1943	1891	1872	1808	1800	1725	1728	1643
A14	15.5	1125	1050	1194	>1950	2015	1976	1947	1899	1880	1822	1812	1745	1744	1668
R02	11.5	*na*	*na*		*na*	>1950		>1950		>1950		>1950		>1950	
R07	16	401	320	465	*na*	2015	2000	1995	1971	1975	1942	1955	1913	1935	1884
R09	15	3857	3771	3947	>1950	2015	1883	1764	1620	1512	1357	1261	1094	1009	831
R10	14	2384	2312	2470	*na*	2015	1927	1850	1750	1685	1574	1520	1398	1354	1221
R11	20	7640	7525	7771	>1950	2015	1821	1639	1432	1263	1044	886	655	510	267
